# Pain Management for Nasogastric Intubation in Pediatrics

**DOI:** 10.7759/cureus.3429

**Published:** 2018-10-09

**Authors:** Shawn Shih, Paul Rosen

**Affiliations:** 1 Internal Medicine, University of Central Florida College of Medicine, Orlando, USA; 2 Pediatrics, Nemours Alfred I. Dupont Children's Hospital, Wilmington, USA

**Keywords:** pain management, nasogastric intubation, pediatrics, distraction, analgesics

## Abstract

Nasogastric (NG) intubation is a common yet one of the most uncomfortable minor procedures done in children and adults alike. A variety of analgesics, such as ketamine, lidocaine, and nitrous oxide, have been shown to reduce pain in various minor pediatric procedures. This retrospective study explores how often various pain management practices are used, either alone or in combination. The study examines NG intubation in pediatrics in one pediatric academic health system. The comfort measures used include analgesics, distraction, child life, swaddling, nitrous oxide, and others. Pharmacological intervention (analgesics) and distraction were most frequently used. Larger randomized studies should be conducted to determine the best practices for comfort measures for NG intubation in order to achieve maximal pain and anxiety reduction for children of various ages.

## Introduction

Nasogastric (NG) intubation is a common procedure in children that involves inserting a thin, hollow (nasogastric) tube through the nose, the throat, and eventually into the stomach. Nasogastric tubes provide access to the stomach for therapeutic and diagnostic purposes. Common indications for nasogastric intubation include gastric decompression, gastrointestinal bleeding, enteral feeding, and administration of radiographic contrast medium or medication, among many others [1]. However, NG intubation causes significant pain and discomfort in patients, children and adults alike.

According to a study in emergency medicine, patients and practitioners have rated NG intubation as the most painful procedure [2]. In order to reduce patient distress, a variety of comfort measures have been tried. Patients requiring NG intubation have been treated with a range of modalities from distraction to analgesia. Analgesics used in various minor pediatric procedures include ketamine, lidocaine, nitrous oxide, and midazolam. Intranasal ketamine has been shown to reduce pain and discomfort in adult patients prior to NG tube insertion, but research is limited in children [3]. Atomized and nebulized lidocaine reduces pain and discomfort in children. However, the delay and distress associated with nebulization may outweigh possible benefits [4].

Despite the availability of various agents for reducing pain for NG tube insertion, pain management is not consistently used in clinical practice in pediatrics [5]. In this study, we analyzed the experience at two pediatric hospitals in an academic children’s health system. The purpose of this study is to determine the frequency of various comfort measures used for pediatric NG intubation. The hope is to understand current practices with the ultimate goal that best practices are developed in the future.

## Materials and methods

In this retrospective study, NG intubation data from 2015 from two pediatric hospitals were analyzed. The database contains methods of pain management used for each patient, if recorded. The database was stripped of all patient identifying information except for patient age and gender. The database contains the dates of tube insertion and removal. Health care professionals who intubated the patients are listed.

Statistical analysis

Frequencies and frequency percentages for each comfort measure at each hospital and both hospitals combined were presented as n (%). The frequencies for the treatment combinations that were used at each hospital were calculated and are presented as n. Since some patients received a combination of comfort measures, the frequencies and frequency percentages do not add up to the total number of patients and 100%, respectively. Comfort measures were not recorded for every patient with NG tube insertion. Only data entries with recorded comfort measures were included in the analysis. Statistical computations were conducted manually.

## Results

The total number of patients between the two hospitals was 130 (Table 1). Pharmacological treatment (64.6%) was the most commonly used comfort measure, followed by distraction/distraction kit (18.5%). Anxiolysis medication (17.7%) was the third most used, although it was not used by Nemours Children’s Hospital (NCH). Though less common, child life (2.31%) and relaxation (6.11%) were used by both hospitals. Swaddling (4.62%), healing touch (2.31%), and nitrous oxide (0.77%) were some of the least common.

**Table 1:** Combined frequency and frequency percentage of pain management modalities used at the two Nemours hospitals.

Pain Management	Combined Total
Pharmacological (Analgesic)	84 (64.6%)
Distraction/Distraction Kit	24 (18.5%)
Anxiolysis Medication	23 (17.7%)
Child Life	3 (2.31%)
Relaxation	8 (6.15%)
Swaddling	6 (4.62%)
Healing Touch	3 (2.31%)
Nitrous Oxide	1 (0.77%)
Other	4 (3.08%)
Total # of Patients	130

Treatment combination

Treatment combinations were used in 8.7% (10/115) and 53.5% (8/15) of the patients at Alfred I. duPont Hospital for Children (AIDHC) and NCH, respectively. The most common combination included pharmacological treatment + distraction/distraction kit at AIDHC and distraction + relaxation + swaddling at NCH. Other combinations were also used, though much less frequent. Pharmacological treatment was used in most of the combinations at AIDHC. Distraction/distraction kit was used in all of the combinations at NCH.

## Discussion

Pain and discomfort associated with NG intubation can be mitigated in many different ways, both pharmacological and non-pharmacological. Pharmacological measures, such as nebulized lidocaine, have been shown to greatly reduce discomfort associated with NG tube in adult patients [6]. However, research does not support nebulized lidocaine for NG intubation in children due to distress associated with nebulization [4]. Alternatively, distraction is a promising alternative as it has been shown to decrease fear, distress, and anxiety in children during painful procedures [7-9]. Distraction has been commonly used in children to relieve pain associated with various procedures and can also lead to reduction of procedure times [10]. A retrospective study of 74,276 procedures at the same two hospitals showed that distraction is more effective at pain reduction than pharmacological and position of comfort for different minor procedures, not including NG intubation [11]. More research is needed to confirm the efficacy of distraction in reducing pain and discomfort associated with NG intubation in children.

Multiple interventions may be combined to achieve more effective pain relief. Pharmacological treatment combined with distraction/distraction kit was the most frequently used combination at AIDHC. This specific combination can be compared with either pharmacological treatment or distraction/distraction kit alone in future research in terms of their pain relief effect. Children in particular are responsive to pain reducing techniques that involve their sense of play, therefore distraction kits containing various toys may be quite effective in reducing procedural pain [12]. Furthermore, the presence of parents or caregivers of pediatric patients during a procedure may help reduce pain, especially if they have been instructed by the medical team to reinforce certain distraction techniques [12].

Despite some differences in the type of comfort measures used between the two hospitals, both pharmacological treatment and distraction were commonly used, likely due to familiarity with these tools/methods, easy accessibility, and/or their efficacy for pain management for other minor pediatric procedures. Some of the other less frequently used interventions in our study, such as nitrous oxide, swaddling, and child life intervention, may also warrant further research, either alone or in combination with other pharmacological and non-pharmacological interventions to test their efficacy for reducing pain associated with NG intubation in children.

Our study has several limitations. As a retrospective chart review, it often provides an inferior level of evidence when compared to prospective studies, for multiple reasons. There were some missing variables that may have provided more insights into our data. Some of these missing variables include the type of analgesic agent used, the method of distraction, patients’ diagnoses, and patients’ level of pain and anxiety before and after the intervention. Furthermore, our sample sizes were skewed between AIDHC (n = 115) and NCH (n = 15), likely due to NCH being a new hospital and lack of data collection. A larger overall sample size that is equally distributed between the two hospitals may be more representative. Nonetheless, the point we want to illustrate is that while data for pain management in pediatric NG intubation is lacking, a variety of comfort measures are being used and further studies are needed to establish higher quality evidence and guidelines.

For future research, we suggest a prospective, controlled trial to test the efficacy of various comfort measures, either alone or in combination. The Wong-Baker Faces Pain Rating Scale has been shown to be a reliable tool for assessing pain in children and would be a great tool for measuring the primary endpoint (pain level) for our purposes [13]. In addition, training of nursing staff by well-trained pediatric anesthesiologists may facilitate successful management of pain in pediatric patients [14].

## Conclusions

In summary, this study reviewed the comfort measures offered in two hospitals in one pediatric health system. Our data demonstrate that there is a wide variability in the types of comfort measures used, with analgesics and distraction being the most commonly used. Larger randomized studies should be conducted to determine the best practices for comfort measures for NG intubation, either alone or in combination, in order to achieve maximal pain and anxiety reduction for children of various ages.
